# Large Haemoperitoneum Caused by a Ruptured Endometrioma: A Case Report

**DOI:** 10.7759/cureus.33113

**Published:** 2022-12-29

**Authors:** Angelos Daniilidis, Georgios Grigoriadis, Panagiotis Papandreou, Dimitra Dalakoura, Alexis Papanikolaou

**Affiliations:** 1 Obstetrics and Gynecology, Aristotle University School of Medicine, Hippokration General Hospital, Thessaloniki, GRC

**Keywords:** laparoscopy, hemorrhage, hemoperitoneum, endometrioma, endometriosis

## Abstract

We report a case of large hemoperitoneum caused by a ruptured endometrioma in a 25-year-old Virgo woman. Hemoperitoneum caused by ruptured endometrioma is a rare entity. The diagnosis should be given consideration when a patient with known or suspected endometriosis presents with signs of intra-abdominal hemorrhage.

## Introduction

Endometriosis is an estrogen-dependent benign disease affecting 2-10% of women of reproductive age and 50% of women with fertility problems. It causes a chronic inflammatory reaction, which results in fibrosis and adhesions [1]. Dysmenorrhea, dyspareunia, dyschezia, dysuria, and intermenstrual pelvic pain are some common clinical manifestations of the disease [2]. We can differentiate three major phenotypes of lesions: superficial peritoneal lesions, ovarian endometriomas, and deep endometriosis (DE). Ovarian endometrioma (commonly known as a ''chocolate cyst'') is a benign cyst of the ovary that contains ectopic endometrial tissue and is a common phenotype of endometriosis (17-44%), with an incidence of approximately 20%-45% in females with infertility [1,3]. Many cases can be misdiagnosed as pelvic inflammatory disease, ectopic pregnancy, and appendicitis [4]. Hemoperitoneum caused by endometriosis is a rare complication, but it can be associated with important morbidity (hypovolaemia, hospital admission for blood transfusion and possible surgical intervention, hypovolaemic shock) [5,6] and even mortality which, according to the literature, can be as high as 11% [7].

We hereby report a case of a 25-year-old Virgo woman with chronic symptoms of endometriosis, who presented with signs of intra-abdominal hemorrhage secondary to endometrioma rupture, with an aim to bring this severe, albeit rare, complication of endometriosis to the attention of clinicians.

## Case presentation

A 25-year-old Virgo woman presented to the emergency outpatient clinic of our department with acute-onset, lower abdominal pain for the past three hours and dizziness. The patient reported a history of chronic dysmenorrhea and polymenorrhea, managed with oral painkillers, with no further diagnostic workup having been undertaken. She was on day eight of her menstrual cycle.

Her vital signs were stable (blood pressure 105/60 mmHg, heart rate 78 beats per minute, body temperature 36.9 °C, oxygen saturation 99%). On physical examination, she was pale and displayed diffuse lower abdominal tenderness with guarding but no rigidity. There were no palpable abdominal masses. The patient did not consent to bimanual or rectal examination or transvaginal ultrasound.

Laboratory tests revealed anemia with a serum hemoglobin level of 8.2 grams per deciliter (g/dL). The rest of her laboratory test results were normal (white blood cells (WBC) 10.100 cells per microliter, C-reactive protein (CRP) of 3 milligrams per liter), and a pregnancy test was negative. A transabdominal ultrasound showed a large pelvic mass and free intra-abdominal fluid. An emergency MRI scan of the lower abdomen and pelvis revealed the presence of a 10 x 10 centimeters adnexal mass, a "kissing ovaries" sign and free fluid, compatible with blood and blood clots, in the pelvic cavity. The uterus, fallopian tubes, and appendix appeared normal.

The patient was admitted, and repeat hemoglobin, performed two hours after admission, was 7.5 g/dl, and two units of packed red blood cells were crossmatched and transfused. Six hours after admission, the patient developed hemodynamic instability (blood pressure 90/55 mmHg, heart rate 108 beats per minute), and an emergency decision for exploratory laparoscopy was made.

**Figure 1:**
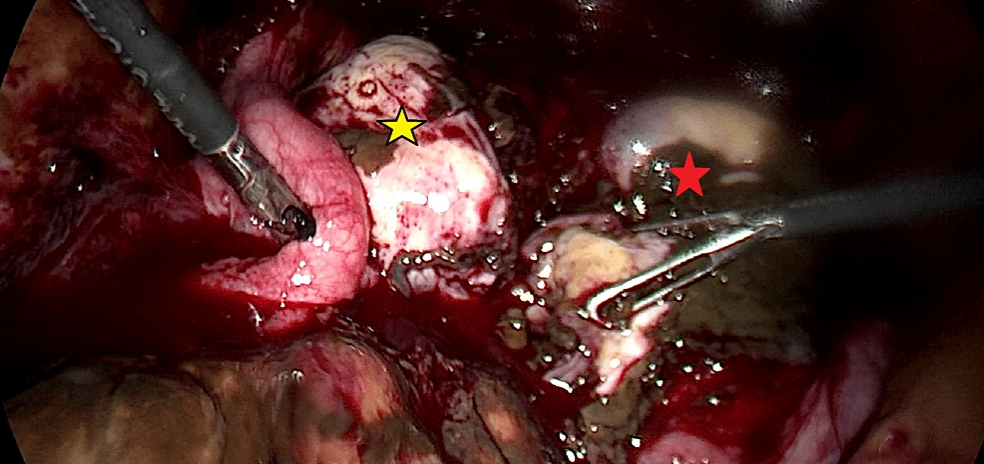
Initial laparoscopic image demonstrating the presence of bilateral ovarian endometriomas with large amount of fresh and clotted blood Red star indicates the right ovary, yellow star indicates the left ovary.

**Figure 2:**
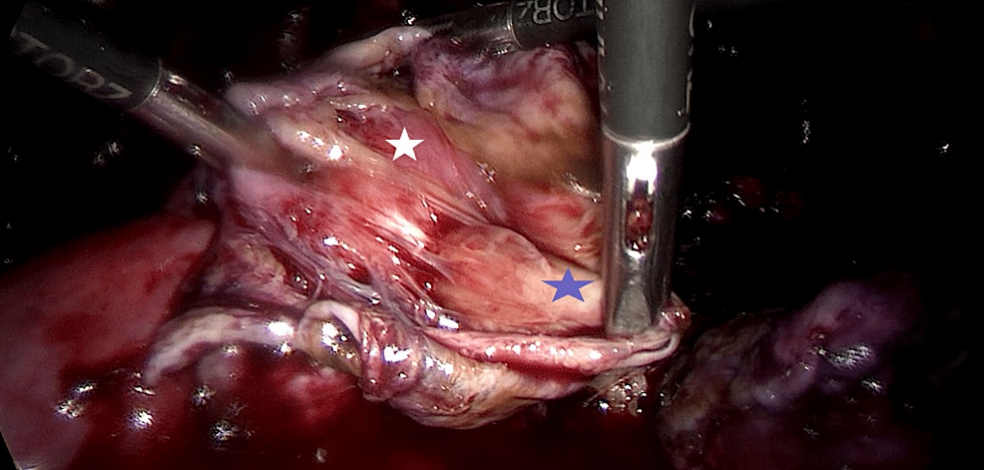
Left ovarian endometrioma excision Blue star indicates the endometrioma cyst wall, white star indicates the ovarian cortex.

**Figure 3:**
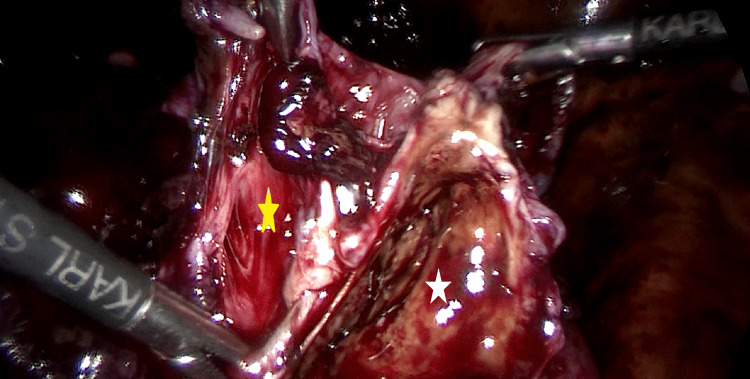
Right ovarian endometrioma excision White star indicates the endometrioma cyst wall, yellow star indicates the ovarian cortex.

Upon entering the abdominopelvic cavity, a large amount of fresh and clotted blood was present. After suctioning the blood (approximately 1700 milliliters), the entire abdominopelvic cavity was explored to identify the source of the bleeding. Bilateral endometriomas were present, and a rupture in the right ovarian cyst wall was recognized, causing hemoperitoneum and free "chocolate" fluid in the abdomen (Figure 1). Lesions of superficial peritoneal endometriosis were identified on the pelvic side walls and pouch of Douglas, and adhesiolysis was performed, mobilizing the ovaries from the posterior uterine wall and the sigmoid colon. The other endometrioma was ruptured during mobilization and its content aspirated. Both cyst capsules were excised (Figure 2, Figure 3), with bipolar diathermy used for hemostasis and cauterization of the peritoneal endometriosis lesions.

The patient received two units of packed red blood cells intraoperatively and two additional units the next day. On discharge, hemoglobin was 10.5 g/dL. Her postoperative course was uneventful and histological analysis of the specimens confirmed the diagnosis of bilateral endometriomas.

## Discussion

Patients with endometriosis present with a variety of symptoms. Endometriosis is usually suspected after evaluating clinical history, physical examination, and imaging studies (ultrasonography and/or magnetic resonance imaging). The gold standard for definitive diagnosis, however, remains visual inspection via laparoscopy and histopathological examination of biopsy specimens [1]. Several theories (retrograde menstruation, endometrial stem cell implantation, Müllerian remnant abnormalities, and coelomic metaplasia) have been proposed to explain the pathogenesis of endometriosis; however, the exact molecular pathway of the disease is still poorly known [2]. Although the exact mechanism behind the formation of ovarian endometrioma remains unclear, it is more likely that pieces of endometrial tissue translocate via the fallopian tubes back to the pelvic cavity and seed onto the ovarian surface, gradually forming into a cyst. During every menstrual cycle, the cyst can grow and undergo high amounts of tension, thus leading to its rupture [8]. Recent data support that approximately 1/3 of patients with ovarian endometrioma (unilateral or bilateral) complain of lower abdominal pain, which is usually induced by the endometriotic cyst rupture and irritation of the peritoneum by the thick contents of the cyst [9]. Hemoperitoneum caused by rupture of endometrioma is a rare entity and is more likely to occur during pregnancy [10]. In addition, endometriosis-related, intra-abdominal hemorrhage may originate from endometriosis lesions at the posterior surface of the uterus and on the utero-ovarian vessels in the parametrium. However, in many cases, the exact source of bleeding cannot be identified [4,5]. Differential diagnosis includes ectopic pregnancy, bleeding from the corpus luteum, ascites originating from malignant tumors, heart or liver disease, and other non-gynecological pathology like appendicitis. Endometriosis-associated hemoperitoneum during pregnancy, which is part of the known entity called spontaneous hemoperitoneum in pregnancy (SHiP), is usually caused by decidualization of the endometriotic lesions and traction by uterine growth [5]. This pathogenetic mechanism is not likely to have caused the rupture of endometriomas in our case.

In our case, the patient had a history of chronic and cyclic dysmenorrhea and presented with acute lower abdominal pain and very low serum hemoglobin in consecutive blood tests. These findings set a strong suspicion for endometriosis-related intra-abdominal hemorrhage. Additionally, urgent imaging (abdominal ultrasound and MRI) revealed bilateral ovarian masses with endometriotic content, no other pelvic pathology, normal appendix, and the presence of free fluid in the abdominal cavity. Ectopic pregnancy was excluded by a negative pregnancy test. 

The gold standard diagnostic and therapeutic tool for this condition is laparoscopy, which allows suctioning of the free abdominal blood, "chocolate" fluid, and blood clots, visualization of the endometriotic cysts, recognition of the rupture and the spot of the active bleeding, resection of the cysts for histopathological diagnosis and control of the hemorrhage with electrocoagulation [11].

## Conclusions

Diagnosis of hemoperitoneum caused by ruptured endometrioma should be given consideration by clinicians when a patient with endometrioma presents with signs of intra-abdominal hemorrhage and after ruling out other possible diagnoses such as ectopic pregnancy, bleeding from the corpus luteum, and non-gynecological pathologies.
